# T85. PRELIMINARY ANALYSES OF THE NEUROCOGNITIVE DATABASE OF PRONIA USING UNIVARIATE STATISTICS: CLINICAL GROUP DIFFERENCES

**DOI:** 10.1093/schbul/sby016.361

**Published:** 2018-04-01

**Authors:** Marco Garzitto, Lana Kambeitz-Ilankovic, Carolina Bonivento, Sara Piccin, Stefan Borgwardt, Eva Meisenzahl, Marlene Rosen, Raimo Salokangas, Rachel Upthegrove, Stephen Wood, Nikolaos Koutsouleris, Paolo Brambilla

**Affiliations:** 1 Scientific Institute IRCCS; 2 Ludwig Maximilian University; 3 University of Udine; 4 University of Basel; 5 Heinrich-Heine University Düsseldorf; 6 University of Cologne; 7 University of Turku; 8 University of Birmingham; 9 Univeristy of Melbourne; 10 University of Milan

## Abstract

**Background:**

Neuro-cognitive deficits are a core feature of psychosis. In the clinical high risk stages of psychosis, neuro-cognitive deficits qualitatively affect the same functions while being quantitatively less marked compared to those in full-blown disorder. Therefore, cognitive impairments are considered to be an important intermediate phenotype for transition to psychosis. Partially overlapping deficits were also reported in depressive disorders, so it is important to identify deficits specifically associated to psychotic symptoms from those common to other conditions. We aimed to identify and differentiate cognitive deficits specifically associated to [i] psychopathology in general (i.e., presence of clinical diagnosis); [ii] psychotic symptoms; [iii] sub- and threshold levels of psychotic symptoms.

**Methods:**

We compared four groups of participants within the project Personalised Prognostic Tools for Early Psychosis Management (PRONIA; www.pronia.eu). The PRONIA Cognitive Battery (PCB) includes 10 tests selected as reliable measures of neuropsychological difficulties in patients at high-risk of psychosis. The scores were obtained from the PRONIA Discovery Sample, which included 707 participants: 278 healthy controls (HC); 138 recent-onset depression (ROD); 139 clinical high-risk (CHR); 152 recent-onset psychosis (ROP), tested in seven sites across Europe. At first the norms were calculated correcting the HC’s raw scores by sex, age, cognitive level, education, and mother language (English, Finnish, German, Italian, or other). Then, univariate analyses of variance with a priori contrasts were used for directly comparing [i] HC vs ROD/CHR/ROP; [ii] ROD vs CHR/ROP; [iii] CHR vs ROP.

**Results:**

The difference in cognitive performance between the clinical groups (ROD, CHR, ROP) as compared to the HC [i], was shown in measures of: speed of execution (ωP2 range 0.016–0.123; all p≤0.035); sustained attention (ωP2: 0.024–0.080; p≤0.022); verbal fluency (ωP2: 0.020–0.031; p≤0.002); emotion recognition (ωP2=0.026; p=0.001); visuo-spatial (ωP2: 0.018–0.049; p≤0.006) and verbal (ωP2: 0.038–0.075; p<0.001) both short- and long-term memory. Three clinical groups did not show significant difference in salience measures when compared with HC (p≥0.053), beyond a main effect of group (ωP2=0.015). Differences between ROD and CHR/ROP groups [ii] were detected in: speed of execution (all p≤0.001); sustained attention (p≤0.011); short-term and working memory (p≤0.004); long-term memory (p≤0.001); semantic verbal fluency (p=0.024); emotion recognition (p=0.005); and estimation of adaptive salience (p=0.021). When compared with ROP, CHR [iii] performed significantly better in the same domains that differentiated ROD from CHR/ROP, with the important exception of long-term memory measures (p≥0.094).

**Discussion:**

These results are consistent with the expectations drawn from previous literature on the neuropsychological impairments in psychotic disorders and CHR participants. Furthermore, PCB showed to be useful in [i] psychopathology in general, [ii] differentiating between recent-onset depression and psychotic symptoms, and [iii] between threshold and sub-threshold psychotic symptoms. Interestingly, long-term memory deficits contributed more in differentiating psychotic symptoms from other psychopathological entities (ROD vs CHR/ROP comparison) than along the spectrum of attenuated psychotic symptoms, resulting in full clinical picture of psychosis (CHR vs ROP). Finally, salience attribution difficulties were confirmed to be associated with (sub-)threshold psychotic symptoms, more than to general psychopathology.

